# Estimating the global prevalence of chronic obstructive pulmonary disease (COPD): a systematic review and meta-analysis

**DOI:** 10.1186/s12889-024-17686-9

**Published:** 2024-01-25

**Authors:** Nadia AL Wachami, Morad Guennouni, Younes Iderdar, Karima Boumendil, Maryem Arraji, Yassmine Mourajid, Fatima Zahra Bouchachi, Mohamed Barkaoui, Mohamed Lahbib Louerdi, Abderraouf Hilali, Mohamed Chahboune

**Affiliations:** 1 Higher Institute of Health Sciences, Laboratory of Sciences and Health Technologies, Hassan First University of Settat, 26000 Settat, Morocco; 2 Higher School of Education and Training, Chouaîb Doukkali University of El Jadida, El Jadida, Morocco; 3 Higher Institute of Sport, Laboratory of Sciences and Health Technologies, Hassan First University of Settat, 26000 Settat, Morocco; 4 Provincial delegation of health, 26000 Settat, Morocco

**Keywords:** Chronic obstructive pulmonary disease, Prevalence, Spirometry, meta-analysis

## Abstract

**Background:**

Chronic obstructive pulmonary disease (COPD) is a major public health problem. The present study aims to provide a global and regional estimate of the prevalence of COPD based on spirometry according to the two most widely used diagnostic criteria of COPD: fixed ratio (FR) and lower limit of normal (LLN).

**Methods:**

We conducted a systematic review of the literature according to PRISMA guidelines. MEDLINE, Web of Sciences, and Scopus databases were searched to identify studies on the spirometry-based prevalence of COPD in individuals aged 40 years and older. The meta-analysis was performed using MedCalc 19 software.

**Results:**

In total, 42 of the 3393 studies reviewed were eligible for inclusion. The overall prevalence of COPD in people aged 40 years and older was 12.64% (95% CI 10.75%-14.65%) and 7.38% (95% CI 5.47% − 9.55%) based on FR and LLN criteria, respectively. By gender, men had a higher prevalence of COPD compared to women (15.47%; 95% CI 12.22%-19.02% for men versus 8.79%; 95% CI 6.94%-10.82% for women). Using the LLN criteria, the prevalence of COPD in both sexes was almost identical (8.67%; 95% CI 8.44%- 8.90% for men and 8.00%; 95% CI 6.42% − 9.73% for women). We reported a high prevalence of COPD among smokers and the elderly by both definitions of airway obstruction. Regional prevalence estimates using the FR definition indicate that the highest COPD prevalence was recorded in the Americas and the lowest was recorded in the Eastern Mediterranean region. Using the LLN definition, the highest prevalence was recorded in the Southeast Asian region and the lowest prevalence was recorded in the American region. The most common COPD stage was stage II, with a prevalence of 50.46%. The results indicate a huge lack of prevalence data in the African and Eastern Mediterranean region. The results were given using a random-effect model due to the high heterogeneity between studies.

**Conclusion:**

Results show that the prevalence of COPD differs according to the diagnostic criteria used. In addition, management and prevention strategies targeting risk factors for COPD are certainly needed to reduce the global burden of this chronic respiratory disease.

**Supplementary Information:**

The online version contains supplementary material available at 10.1186/s12889-024-17686-9.

## Background

Chronic obstructive pulmonary disease (COPD) is defined as a heterogeneous lung condition characterized by chronic respiratory symptoms (dyspnea, cough, and expectoration) due to persistent abnormalities of the airways and/or alveoli that often result in progressive airflow limitation [1]. It represents a real challenge for global health systems, with significant socioeconomic and health consequences [2]. According to the World Health Organization (WHO), COPD is considered among the top ten causes of death worldwide [3]. In 2016, 251 million cases of COPD were recorded worldwide, according to estimates from the Global Burden of Disease (GBD) study [4]. It imposes a massive burden, mainly due to the high cost and its negative impact on the quality of life of affected patients [5]. COPD is an important cause of mortality. Between 2009 and 2019, the mortality rate of COPD increased by 35.4% [6]. Furthermore, WHO mortality and disease burden projections state that COPD will be the third leading cause of death worldwide by 2030 [7].

COPD is a multifactorial disease, with tobacco smoke being the best-known and most important risk factor for irreversible airflow obstruction [8, 9]. However, non-smokers can also develop COPD. Estimates suggest that 25–45% of COPD cases are non-smokers [10]. Outdoor and indoor air pollution from biomass smoke, occupational exposures to dust and chemical gases in the workplace, male gender, advanced age, low body mass index, history of respiratory diseases, and family history of respiratory diseases are all factors listed as having a role in the development of COPD in non-smokers [11, 12].

Worldwide, COPD remains an underestimated and underdiagnosed disease. The main causes of underdiagnosis identified in the literature are lack of knowledge of the disease on the part of patients and physicians, underestimation of symptoms, and underuse of the spirometer to establish the diagnosis [13]. Indeed, the spirometer is the gold standard for the diagnosis of COPD [14, 15]. The most frequently used diagnostic criteria are the fixed ratio (FR), which states that the presence of a ratio of forced expiratory volume in one second (FEV1) to forced vital capacity (FVC) post-bronchodilator less than 0.70 confirms the diagnosis, and the lower limit of normal (LLN) criteria, in which the diagnosis is based on the comparison of values measured by spirometry with reference values identified from healthy and non-smoking subjects [1, 16]. Non-use of pulmonary function testing and over-reliance on clinical diagnosis can lead to misdiagnosis of airflow obstruction, as evidenced by data from the published literature, which showed that spirometry assessment revealed that 43.8% of cases diagnosed by a physician were misdiagnosed [17].

Estimating the global prevalence of COPD is crucial to understanding its magnitude and reducing the burden of disease associated with this chronic condition. Systematic reviews and meta-analyses of global COPD prevalence estimates exist in the literature. For example, Adeloye et al. reported the summary global prevalence of COPD without considering the case definitions used [18]. In addition, Vermaghani and colleagues estimated the prevalence of COPD based only on studies using the fixed ratio as the diagnostic criteria [19]. One study estimated the worldwide prevalence of COPD using the FR and LLN criteria [20]. The main objective of this review is to provide a recent estimate of the global and regional prevalence of COPD according to the FR and LLN criteria over the past 6 years. Our study also aims to estimate the prevalence of COPD according to several parameters, such as gender, severity stage, age groups, and smoking status, using both diagnostic criteria. These estimates will serve as a basis for understanding the burden of COPD worldwide and for developing effective prevention and management strategies to address it.

## Materials and methods

This review was developed according to the guidelines of the Preferred Reporting Items for Systematic Reviews and Meta-analyses (PRISMA) [21] (Additional File 1).

### Inclusion criteria

The inclusion criteria in this systematic literature review included (i) studies reporting the prevalence of COPD based on spirometry testing; (ii) studies reporting the prevalence of COPD in people aged 40 years and older based on spirometry testing; (iii) studies published between 2016 and 2022; (iv) publications published in English and French; and (v) studies in open access.

### Information source

We conducted a literature search of the MEDLINE, Web of Sciences, and Scopus databases to identify relevant studies related to the research objective and published between January 2016 and July 2022, using the following keywords: COPD, prevalence, and epidemiology. A combination of keywords using search operators was performed to refine the search results and identify relevant publications.

### Studies selection

The studies identified by the literature search were first selected on the basis of their titles and abstracts. If there was uncertainty about the eligibility of a study, a second selection was made by consulting the full text. Studies that did not meet the eligibility criteria were excluded from the analysis. Reference lists of the selected studies and related reviews of literature were manually checked for potential inclusions. The study selection process was performed by two postgraduate students and two professors.

### Data extraction

To extract data from the included studies, a data extraction form was created on Microsoft Excel. The form included; (a) title; (b) first author’s name; (c) journal name; (d) publication year; (e) publication language; (f) study design; (g) study location; (h) study objective; (i) data collection tools; (j) COPD diagnostic criteria; k) results found; and l) the author’s observations and conclusions.

### Quality assessment of included studies

To assess the quality of the studies included in this review, we used the STROBE quality assessment checklist (Strengthen The Reporting of Observational Studies in Epidemiology) [22–24]. The assessment was based on five criteria: study objective, sampling technique, sample size, measurement of lung function, and diagnostic criteria used.

When the study objective was well defined, we assigned a score of 1, otherwise,a score of 0 was assigned. If the study used a random sampling technique, we gave it a score of 1. If the study used a non-random sampling technique or did not mention the technique used, we gave it a score of 0. If the sample size was greater than 384 participants and/or the calculation was well defined, we gave a score of 1, otherwise, a score of 0 was given. Regarding the assessment of lung function, we assigned a score of 1 for all studies since we only included studies reporting the prevalence of COPD based on a spirometry test. We assigned a score of 2 if the diagnosis of COPD was made on the basis of the FR criteria. When the LLN criteria were used, a score of 1 was assigned. A total score of 6 points indicates that the study is of high quality. When a score of 4 or 5 points has been obtained, the study is considered to be of moderate quality. Lower scores of 3, 2, 1 and 0 indicate that the study is of low quality.

### Data synthesis and analysis

Given the high level of heterogeneity, a rondom effect meta-analysis was performed. The choice of meta-analysis model (random effect, fixed effect, or mixed effect) was determined by the existence or presence of heterogeneity between the included studies. Inter-study heterogeneity was measured by the I^2^ test to estimate the percentage of variability between the included studies [25, 26]. An I^2^ value > 70% indicates high heterogeneity. Heterogeneity is said to be moderate if the I^2^ value is between 70 and 50%. An I^2^ < 50% indicates low heterogeneity between the results of the studies [27, 28]. Forest plots were based on the prevalence of COPD according to the two diagnostic criteria FR and LLN. A meta-regression was performed to provide COPD estimates of COPD by several parameters and to detect sources of heterogeniety. A sensitivity analysis was performed to see the effect of studies with a high weight on the overall results of the meta-analysis. The meta-analysis was performed using MedCalc version 19.4 statistical software (MedCalc Software bv; https://www.medcalc.org; 2019). Egger’s test is used to assess the risk of bias between studies while visualizing the symmetry or asymmetry of the funnel plots.

## Results

### Studies selection

**Fig. 1:**
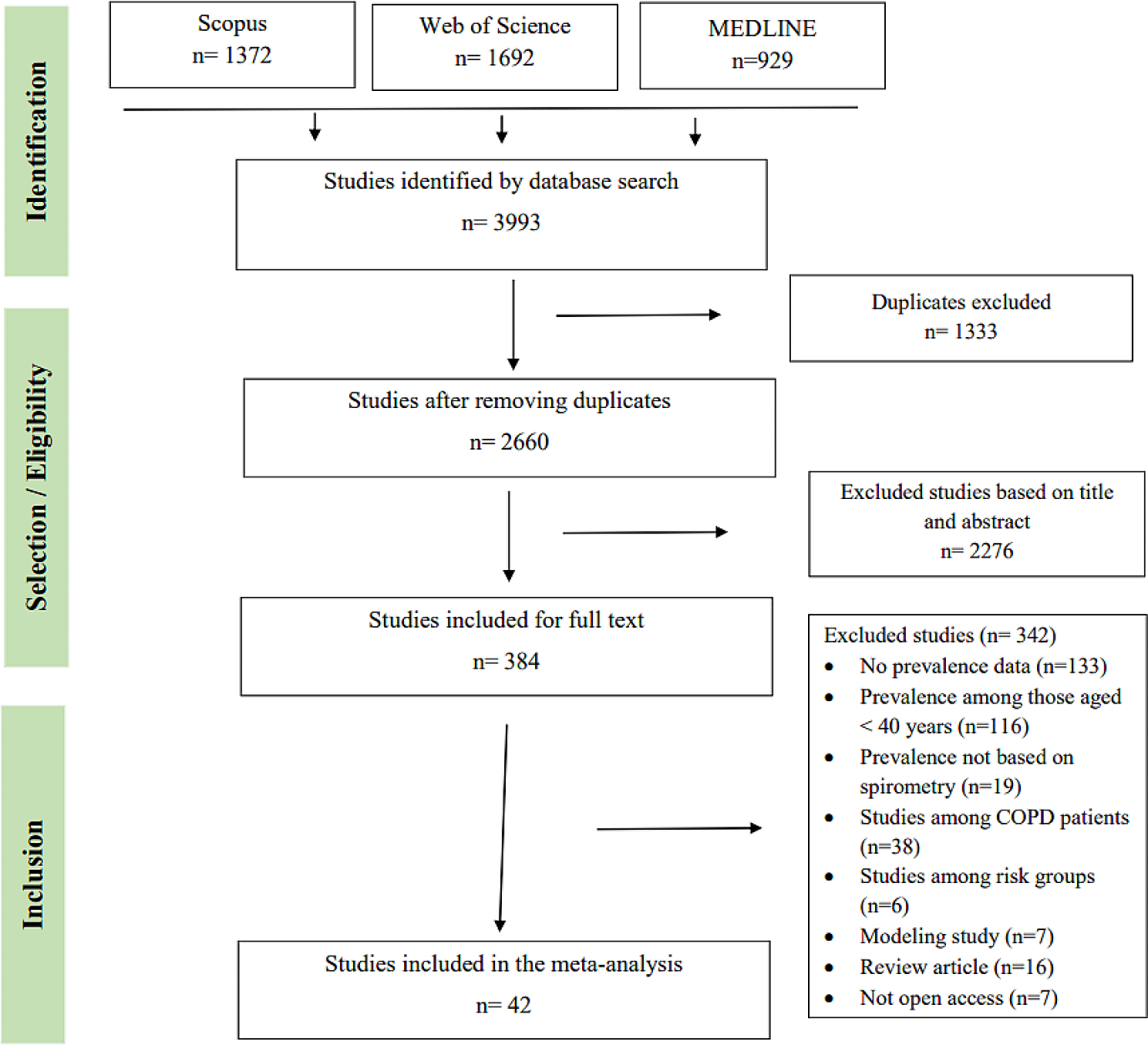
Flow chart of the search and selection steps of the included studies

A total of 3993 potentially relevant records were identified through a database search (1372 studies identified from Scopus, 1692 identified from the Web of Sciences, and 929 identified from MEDLINE). After deleting 1333 duplicates, 2276 were excluded on the basis of their titles and abstracts. Full-text reading of 384 articles excluded 342 that did not meet the eligibility criteria. Overall, 42 studies meeting the inclusion criteria were included in this systematic review of the literature and meta-analysis (Fig. 1).

### Characteristics of the included studies

All of the studies included in the review were published in the English language. Seven studies were published in 2022, seven in 2021, seven in 2020, four in 2019, four in 2018, four in 2017, and nine studies were published in 2016. Thirty-seven studies used a cross-sectional design while a longitudinal design was used by only five studies. Eligible studies were conducted in twenty-three countries. Twenty-three studies were conducted in the Western Pacific region, eight in the European region, four in the Americas region, three in the Southeast Asian region, three in the Eastern Mediterranean region, and only one study was conducted in the African region. Regarding the study area, thirty-one studies were conducted in a mixed area, nine in urban areas, and two in rural areas. The total sample size was 339475 participants aged 40 years and older and ranged from 141 to 94551 participants. The mean age of the participants was 57.30 years and ranged from 44 to 68 years. To make the diagnosis of COPD, twenty-four studies used the FR, criteria and six studies used the LLN criteria. Twelve studies made the diagnosis of persistent obstruction using both criteria at the same time (Table 1).

**Table 1:** Characteristics of the studies included in the review on the prevalence of COPD

Author / Year of publication	Type of study	Study location	Study setting	Sample size average	Age in years	Diagnostic criteria for COPD
Bourbeau et al., 2022 [29]	Prospective longitudinal study	Canada	Mixed	1452	66.6	FR
Farooqi et al.,2022 [30]	Cross-sectional study	Canada	Mixed	21,242	64	LLN
Grafino et al.,2022 [31]	Cross-sectional study	Lisbon/ Portugal	Mixed	241	60	FR and LLN
Machiguchi et al.,2022 [32]	Retrospective epidemiological study	Japan	Mixed	4364	53	FR
Pagano et al.,2022 [33]	Cross-sectional study	Australia	Mixed	141	68	FR
Wang et al.,2022 [34]	Cross-sectional study	China	Mixed	33,664	55.4	FR
Xiao et al.,2022 [35]	Cross sectional study	Guangdong/ China	NA	202	58.2	FR
Kim et Kang.,2021 [36]	Cross-sectional study	South Korea	Mixed	12,919		LIN
Leung et al.,2021 [37]	Cross-sectional study	Canada	Urban	4893	56.6	FR and LLN
Li et al.,2021 [38]	Cross-sectional study	Kashi / China	Rural	2963	55.4	FR
Shangguan et al..,2021 [39]	Cross-sectional study	Liaoning/ China	Mixed	2194	-	FR
Su et al.,2021 [40]	Cross-sectional population-based study	Jiangsu / China	Mixed	3407	57.2	FR
Tamaki et al.,2021 [41]	Cross-sectional observational study	Okinawa / Japan	Mixed	2518	52	FR
Zhang et al.,2021 [42]	Cross-sectional study	Jiangsu / China	Mixed	2421	56.63	FR
Adhikari et al.,2020 [43]	Cross-sectional study	Pokhara / Nepal	Semi urban	1438	55	FR and LLN
Kim et al.,2020 [44]	Longitudinal study	South Korea	Mixed	6341	51.3	FR
Melbye et al.,2020 [45]	Cross-sectional study	Tromsø / Norway	Mixed	7247	63	LLN
Sharifi et al.,2020 [46]	Cross-sectional study	Mazandaran / Iran	Mixed	1007	44	FR
Sumit et al.,2020 [47]	Cross-sectional study	Bangladesh	Mixed	373	-	FR
Timur et al.,2020 [48]	Cross-sectional study	Kayseri / Turkey	Mixed	386	53.3	FR
Yan et al.,2020 [49]	Cross-sectional study	Suzhou / China	Mixed	4725	62.2	FR
Bikbov et al.,2019 [50]	Cross-sectional study	Ufa / Russia	Mixed	5392	59	FR and LLN
De Matteis et al.,2019 [51]	Cross-sectional study	United Kingdom	Urban	94,551	55.9	LLN
Sheng et al.,2019 [52]	Cross-sectional study	Ningbo / Chin	Rural	1371	-	FR
Zha et al.,2019 [53]	Cross-sectional study	Anhui / China	Mixed	2770	53.8	FR and LLN
Broström et al.,2018 [54]	Cross-sectional study	Tartu in Estonia, Reykjavik in Iceland, and Uppsala in Sweden/Nordic Baltic Region	Urban	1993	58.73	LLN
Ding et al.,2018 [55]	Cross-sectional study	Hlai / China	Mixed	5637	-	FR
Fang et al..,2018 [56]	National cross-sectional study	China	Mixed	66 752	54.9	FR
Leem et al.,2018 [57]	Prospective cohort study	Ansunge and Ansan/Korea	Mixed	6517	-	FR
Kotaki et al.,2017 [58]	Cross-sectional study	Omuta / Japan	Urban	293	67.65	FR
Nakao et al.,2017 [59]	Cross-sectional study	Oulan Bator / Mangolia	Mixed	746	54.1	FR
Sobrino et al. 2017 [60]	Longitudinal prospective cohort study	Latin America (Bariloche and Marcos Paz, Argentina; Temuco, Chile; and Pando-Barros Blancos,Uruguay)	Mixed	4354	-	FR and LLN
Torén et al.,2017 [61]	Cross-sectional validation study	Sweden	Mixed	1050	-	FR and LLN
Denguezli et al.,2016 [62]	Cross-sectional study	Sousse / Tunisia	Mixed	661	52	FR and LLN
El Rhazi et al.,2016 [63]	Cross-sectional study	Fez / Morocco	Urban	768	-	FR and LLN
Fukuyama et al.,2016 [64]	Cross-sectional study	Hisayama / Japan	Mixed	2232	61.1	FR
Karrasch et al.,2016 [65]	Observational cross-sectional study	Augsburg. Germany	Mixed	2256	61.6	FR and LLN
Koul et al.,2016 [66]	Cross-sectional study	Kashmir / India	Mixed	953	51.62	FR and LLN
Loh et al.,2016 [67]	Cross-sectional study	Penang / Malaysia	Sub urban	663	-	FR and LLN
Obaseki et al.,2016 [68]	Cross-sectional study	Ile-Ife / Nigeria	Sub urban	875	-	LLN
Omori et al. 2016 [69]	Cross-sectional study	Japan	Mixed	22,293	54.7	FR
Park et al. 2016 [70]	Cross-sectional study	Korea	Mixed	3283	59.35	FR

COPD: chronic obstructive pulmonary disease; FR: fixed ratio; LLN: lower limit of normal

### Quality of included studies

Of the forty-two eligible studies, twenty-one were of high quality, and twenty-one were of moderate quality. No study was of low quality. Table 2 shows the scores awarded according to each evaluation criteria and the total score for each study.

**Table 2:** Quality of included studies

Study	Study purpose	Sampling technique	Sample Size	Assessement of lung function	COPD diagnostic criteria	Total score	Study quality
Bourbeau et al. 2022 [29]	Clearly defined (1)	Hazards sampling (1)	*n* = 1452 (1)	Objective (1)	FR (2)	6	high quality
Farooqi et al. 2022 [30]	Clearly defined (1)	Stratified random (1)	21 242 (1)	Objective (1)	LLN (1)	5	moderate quality
Grafino et al. 2022 [31]	Clearly defined (1)	Not mentioned (0)	241 (0)	Objective (1)	FR and LLN (2)	4	moderate quality
Michiguchi et al. 2022 [32]	Clearly defined (1)	Not mentioned (0)	*n* = 4364 (1)	Objective (1)	FR (2)	5	moderate quality
Pagano et al. 2022 [33]	Clearly defined (1)	Not mentioned (0)	141 (0)	Objective (1)	FR (2)	4	moderate quality
Wang et al. 2022 [34]	Clearly defined (1)	Hazards sampling (1)	*n* = 33 664 (1)	Objective (1)	FR (2)	6	high quality
Xiao et al. 2022 [35]	Clearly defined (1)	Randomized (1)	*n* = 202 (0)	Objective (1)	FR (2)	5	moderate quality
Kim et Kang 2021 [36]	Clearly defined (1)	Multi-stage complex randomness (1)	*n* = 12 919 (1)	Objective (1)	LLN (1)	5	moderate quality
Leung et al. 2021 [37]	Clearly defined (1)	Rondomized (1)	*n* = 4893 (1)	Objective (1)	FR and LLN (2)	6	high quality
Li et 2021 [38]	Clearly defined (1)	Random in clusters (1)	*n* =2963 (1)	Objective (1)	FR (2)	6	high quality
Shanggaun et al. 2021 [39]	Clearly defined (1)	Multi-stage randomness (1)	*n* =2194 (1)	Objective (1)	FR (2)	6	high quality
Su et al. 2021 [40]	Clearly defined (1)	Multi-stage randomness (1)	*n* =3407 (1)	Objective (1)	FR (2)	6	high quality
Tamaki et al. 2021 [41]	Clearly defined (1)	Non-random (0)	*n* = 2518 (1)	Objective (1)	FR (2)	5	moderate quality
Zhang et al. 2021 [42]	Clearly defined (1)	Multi-stage randomness (1)	*n* = 2421 (1)	Objective (1)	FR (2)	6	high quality
Adhikari et al. 2020 [43]	Clearly defined (1)	Systemic randomness (1)	*n* =1438 (1)	Objective (1)	FR and LLN (2)	6	high quality
Kim et al. 2020 [44]	Clearly defined (1)	Randomized (1)	*n* =6341 (1)	Objective (1)	FR (2)	6	high quality
Melbye et al. 2020 [45]	Clearly defined (1)	Randomized (1)	*n* =7247 (1)	Objective (1)	LLN (1)	5	moderate quality
Sharifi et al. 2020 [46]	Clearly defined (1)	Randomized (1)	*n* =1007 (1)	Objective (1)	FR (2)	6	high quality
Sumit et al. 2020 [47]	Clearly defined (1)	Not mentioned (0)	*n* = 373 (1)	Objective (1)	FR (2)	5	moderate quality
Timur et al. 2020 [48]	Clearly defined (1)	Not mentioned (0)	*n* = 386 (1)	Objective (1)	FR (2)	5	moderate quality
Yan et al. 2020 [49]	Clearly defined (1)	Non-random (0)	*n* =4725 (1)	Objective (1)	FR (2)	5	moderate quality
Bikbov et al. 2019 [50]	Clearly defined (1)	Randomized (1)	*n* =5392 (1)	Objective (1)	FR and LLN (2)	6	High quality
De Matteis et al. 2019 [51]	Clearly defined (1)	Randomized (1)	*n* =94,551 (1)	Objective (1)	LLN (1)	5	moderate quality
Sheng et al. 2019 [52]	Clearly defined (1)	Non-random (0)	*n* = 1371 (1)	Objective (1)	FR (2)	5	moderate quality
Zha et al. 2019 [53]	Clearly defined (1)	Random, complex in several degrees (1)	*n* =2770 (1)	Objective (1)	FR and LLN (2)	6	high quality
Brostrôm et al. 2018 [54]	Clearly defined (1)	Randomized (1)	*n* = 1993 (1)	Objective (1)	LLN (1)	5	moderate quality
Ding et al. 2018 [55]	Clearly defined (1)	Not mentioned (0)	*n* =5637 (1)	Objective (1)	FR (2)	5	moderate quality
Fang et al. 2018 [56]	Clearly defined (1)	Random, complex in several degrees (1)	*n* = 66,752 (1)	Objective (1)	FR (2)	6	high quality
Leem et al. 2018 [57]	Clearly defined (1)	Randomized clustering in two stages (1)	*n* =6517(1)	Objective (1)	FR (2)	6	high quality
Kotaki et al. 2017 [58]	Clearly defined (1)	Not mentioned (0)	*n* = 293 (0)	Objective (1)	FR (2)	4	moderate quality
Nakaao et al. 2017 [59]	Clearly defined (1)	Non-random (0)	*n* = 746 (1)	Objective (1)	FR (2)	5	moderate quality
Sobrino et al. 2017 [60]	Clearly defined (1)	Random, stratified at four degrees (1)	*n* =4345 (1)	Objective (1)	FR and LLN (2)	6	high quality
Torén et al. 2017 [61]	Clearly defined (1)	Rondomized (1)	*n* = 1050 (1)	Objective (1)	FR and LLN (2)	6	high quality
Denguizli et al. 2016 [62]	Clearly defined (1)	Stratified random (1)	*n* = 661 (1)	Objective (1)	FR and LLN (2)	6	high quality
El Rhazi et al. 2016 [63]	Clearly defined (1)	Randomized (1)	*n* =768 (1)	Objective (1)	FR and LLN (2)	6	high quality
Fukuyama et al. 2016 [64]	Clearly defined (1)	Not mentioned (0)	*n* = 2232 (1)	Objective (1)	FR (2)	5	moderate quality
Karrash et al. 2016 [65]	Clearly defined (1)	Not mentioned (0)	*n*= 2256 (1)	Objective (1)	FR and LLN (2)	5	moderate quality
Koul et al. 2016 [66]	Clearly defined (1)	Randomized (1)	*n* =757 (1)	Objective (1)	FR and LLN (2)	6	high quality
Loh et al. 2016 [67]	Clearly defined (1)	Random simple stratified (1)	*n* = 663 (1)	Objective (1)	FR and LLN (2)	6	high quality
Obaseki et al. 2016 [68]	Clearly defined (1)	Random in three stages (1)	*n* = 875 (1)	Objective (1)	LLN (1)	5	moderate quality
Omori et al. 2016 [69]	Clearly defined (1)	Not mentioned (0)	*n* =22,293 (1)	Objectif (1)	FR (2)	5	moderate quality
Park et al. 2016 [70]	Clearly defined (1)	Random, complex in several degrees (1)	*n* = 3283 (1)	Objective (1)	FR (2)	6	high quality

COPD : chronic obstructive pulmonary disease ; FR : fixed ratio ; LLN : lower limit of normal

### Estimated overall prevalence of COPD

**Fig. 2:**
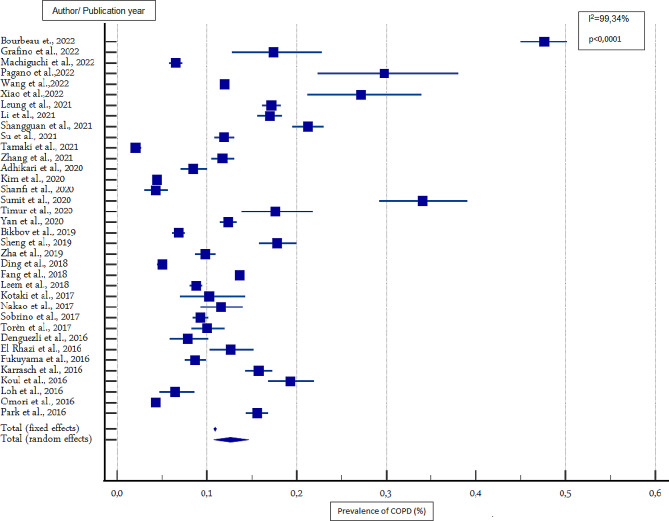
Forest representation of the prevalence of COPD according to the FR criteria

**Fig. 3:**
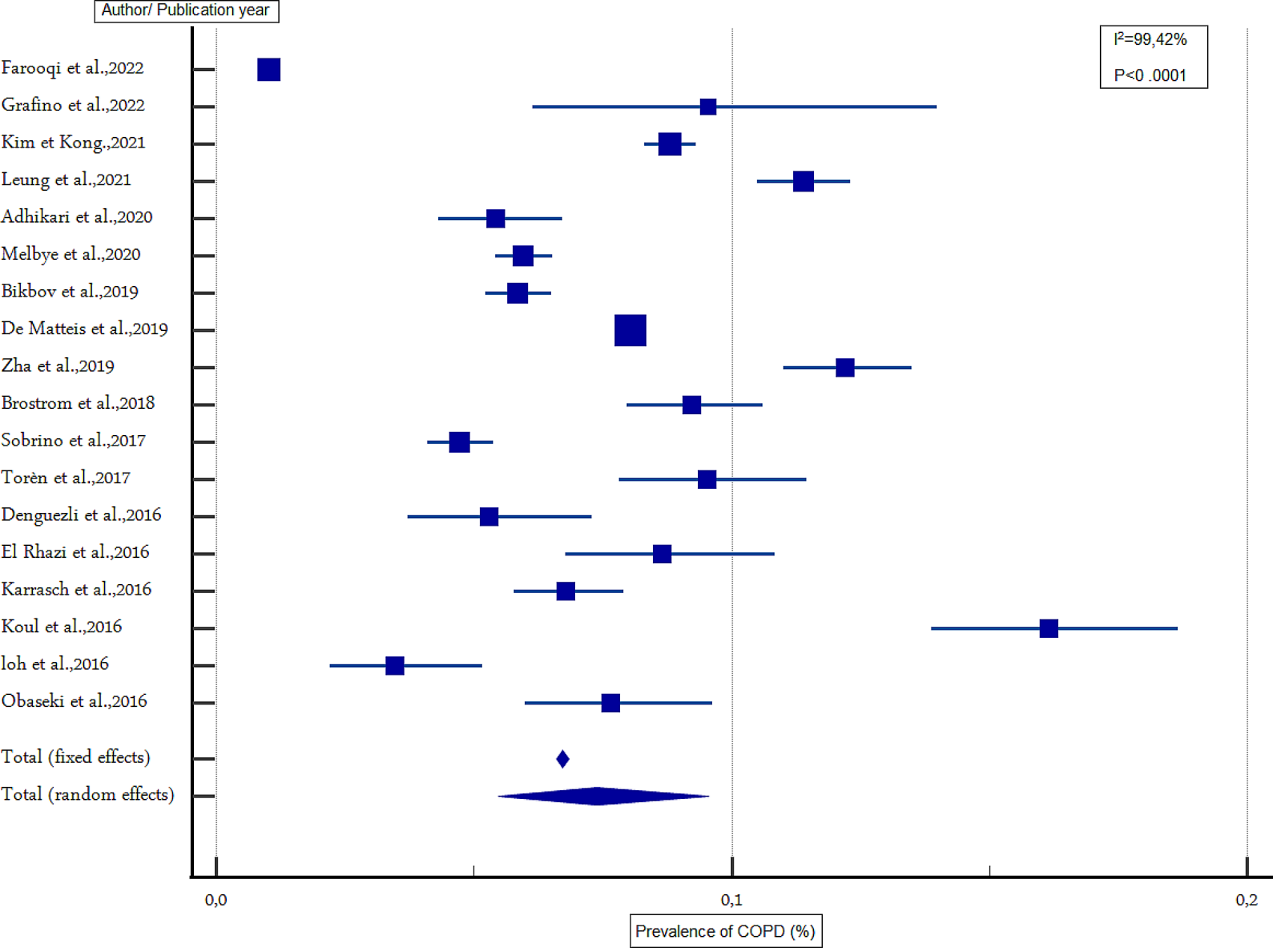
Forest representation of the prevalence of COPD according to the LLN criteria

The overall prevalence of COPD in people aged 40 years and above was 12.64% (95% CI 10.75%-14.65%) and 7.38% (95% CI 5.47% − 9.55%) based on FR and LLN criteria, respectively (Table 3; Figs. 2 and 3). According to the FR criteria, men had a higher prevalence compared to women (15.47%, 95% CI 12.22% − 19.02% and 8.79%, 95% CI 6.94% − 10.82% among men and women, respectively). According to the LLN criteria, there was no difference in the prevalence of COPD between the two sexes (the prevalence in men and women was 8.67% (95% CI 8.44% − 8.90%) and 8.00% (95% CI 6.42% − 9.73%), respectively) (Table 2). The most common stage of irreversible airflow obstruction was stage II, with a prevalence of 50.46% (95% CI 44.59% − 56.33%) (*p* < 0.0001), followed by stage I, with a prevalence of 35.21% (95% CI 26.70%- 44.23%) (*p* < 0.0001). Stages III and IV were the least frequent, with a prevalence of 6.77% (95% CI 4.78% − 9.07%) (*p* < 0.0001) and 1.047% (95% CI 0.60%-1.60%) respectively (*p* < 0.0003). Sensitivity analysis showed no change in the overall prevalence of COPD according to the two diagnostic criteria.

**Table 3:** Overall and gender-specific prevalence of COPD among individuals aged 40 years and older (combined crude prevalence of COPD estimated from 42 studies included in the analysis)

		FR criteria	LLN criteria
Both genders	Total number of studies	36	18
	Total number of participants	200,723	164,184
	Prevalence of COPD (%) (95% CI)	12.64 (10.75–14.65)	7.38 (5.47–9.553)
	I^2^ (%)	99.34	99.42
	p value	*P* < 0.0001	*P* < 0.0001
Men	Total number of studies	29	13
	Total number of participants	77,353	57,583
	Prevalence of COPD (%) (95% CI)	15.47 (12.22–19.02)	8.67 (8.44–8.90)
	I^2^ (%)	99.29	94.75
	*p* value	*P* < 0.0001	*P* < 0.0001
Women	Total number of studies	29	13
	Total number of participants	74,597	57,583
	Prevalence of COPD (%) (95% CI)	8.79 (6.94- 10.828)	8.001 (6.42–9.73)
	I^2^ (%)	98.58	96.00
	*p* value	*P* < 0.0001	*P* < 0.0001

COPD: chronic obstructive pulmonary disease; FR: fixed ratio; LLN: lower limit of normal

### Estimated overall prevalence of COPD among individuals over 40 years of age by age category and smoking status

The random-effects meta-analysis indicated a significant increase in the prevalence of COPD as the population aged, independent of the diagnostic criteria employed. By the fixed ratio criteria, the prevalence of COPD increased from 4.37% (95% CI 2.76% − 6.33%) in those aged 40–49 years to 24.03% (95% CI 20.04%-28.26%) in those aged 70 years and older. By LLN criteria, the prevalence was 5.22% (95% CI 2.34%-9.17%) and 14.23% (95% CI 11.96%-16.75%) in those aged 40–49 and 70 years and older, respectively (Table 4).

Smokers had a higher prevalence of COPD than non-smokers. Using the FR criteria, the prevalence of COPD among never smokers, former smokers, and current smokers was 8.15%, 18.38%, and 21.51%, respectively. Using the LLN criteria, the respective prevalence of irreversible airflow obstruction among never smokers, former smokers, and current smokers were 3.77%, 7.55%, and 11.13% (Table 4).

**Table 4:** Overall prevalence of COPD by age category and smoking status

FR criteria				
Age group	Total number of participants	Prevalence of COPD % (95% CI)	I^2^ (%)	*p* value
40–49 years	18,287	4.37 (2.76–6.33)	96.46	*P* < 0.0001
50–59 years	16 362	9.54 (6.70-12.82)	97.21	*P* < 0.0001
60–69 years	11,920	15.84 (11.85–20.29)	97.04	*P* < 0.0001
70 years and older	5923	24.03 (20.04–28.26)	91.60	*P* < 0.0001
Smoking status				
Never smokers	55,903	8,154 (5.569–11.180)	98.59	*P* < 0.001
Former smokers	11,484	18,385 (12.035–25.731)	98.42	*P* < 0.001
Currently smokers	26,370	21.512 (16.394–27.118)	97.93	*P* < 0.0001
**LLN criteria**				
Age group	Total number of participants	Prevalence of COPD % (95% CI)	I^2^ (%)	p value
40–49 years	2715	5.22 (2.34–9.17)	93.02	*P* < 0.0001
50–59 years	2173	6.61 (2.05–13.51)	96.39	*P* < 0.0001
60–69 years	1478	14.29 (12.54–16.17)	78.95	0.0002
70 years and older	852	14.23 (11.96–16.75)	79.49	0.0002
Smoking status				
Never smokers	69,308	3.778 (0.369–10.526)	99.81	*P* < 0.001
Former smokers	42,159	7.550 (2.989–13.962)	99.46	*P* < 0.001
Currently smokers	7575	11.135 (5.019–19.271)	98.14	*P* < 0.001

COPD : chronic obstructive pulmonary disease ; FR : fixed ratio ; LLN : lower limit of normal

### Estimated regional prevalence of COPD among people aged 40 years and above

According to the FR criteria, the highest prevalence was recorded in the American region with a proportion of 22.93%, followed by the South East Asian region with an estimate of 19.48%. This was followed by the Europe, Western Pacific, and Eastern Mediterranean regions with a proportion of 13.09%, 11.17%, and 7.95%, respectively. No studies were found on the prevalence of COPD using the FR criteria in the African region. Using the LLN criteria, the South East Asian region recorded the highest prevalence of 10.17%, followed by the African region with an estimate of 7.7%, the Western Pacific region with a proportion of 7.56%, the European region with a proportion of 7.34%, and the Eastern Mediterranean region with a prevalence of 6.9%. The American region recorded the lowest prevalence (4.82%) (Table 5).

**Table 5:** Prevalence of COPD among people aged 40 years and older by WHO geographical region

WHO region	FR Criteria			LLN criteria		
	Prevalence of COPD (%) (95% CI)	I^2^ (%)	*P* value	Prevalence of COPD (%) (95% CI)	I^2^ (%)	*p* value
EMR	7.95 (3.72–13.59)	95.24	*P* < 0.0001	6.9 (0.042–0.110)	83.34	0.000
EUR	13.09 (8.32–18.76)	97.63	*P* < 0.0001	7.34 (6.7–8.01)	95.86	*P* < 0.0001
AMR	22.93 (8.18–42.34)	99,77	*P* < 0.0001	4.82 (0.49–13.23)	99.80	*P* < 0.0001
SEAR	19.48 (7.99–34.47)	98.65	*P* < 0.0001	10.17 (2.22–22.94)	98.63	*P* < 0.0001
WPR	11.17 (9.11–13.42)	99.42	*P* < 0.0001	7.56 (3.70-10.11)	94.96	*P* < 0.0001
AFR	-			7.7 ( 6.0–9.8)	-	*P* < 0.0001

EMR: Eastern Mediterranean Region; EUR: European Region; AMR: Americas Region; SEAR: South East Asia Region; WPR: Western Pacific Region; AFR: African Region; COPD: chronic obstructive pulmonary disease; FR: fixed ratio; LLN: lower limit of normal

### Estimated overall prevalence of COPD among individuals aged 40 years and older during the period 2016–2019 and 2020–2022

**Fig. 4:**
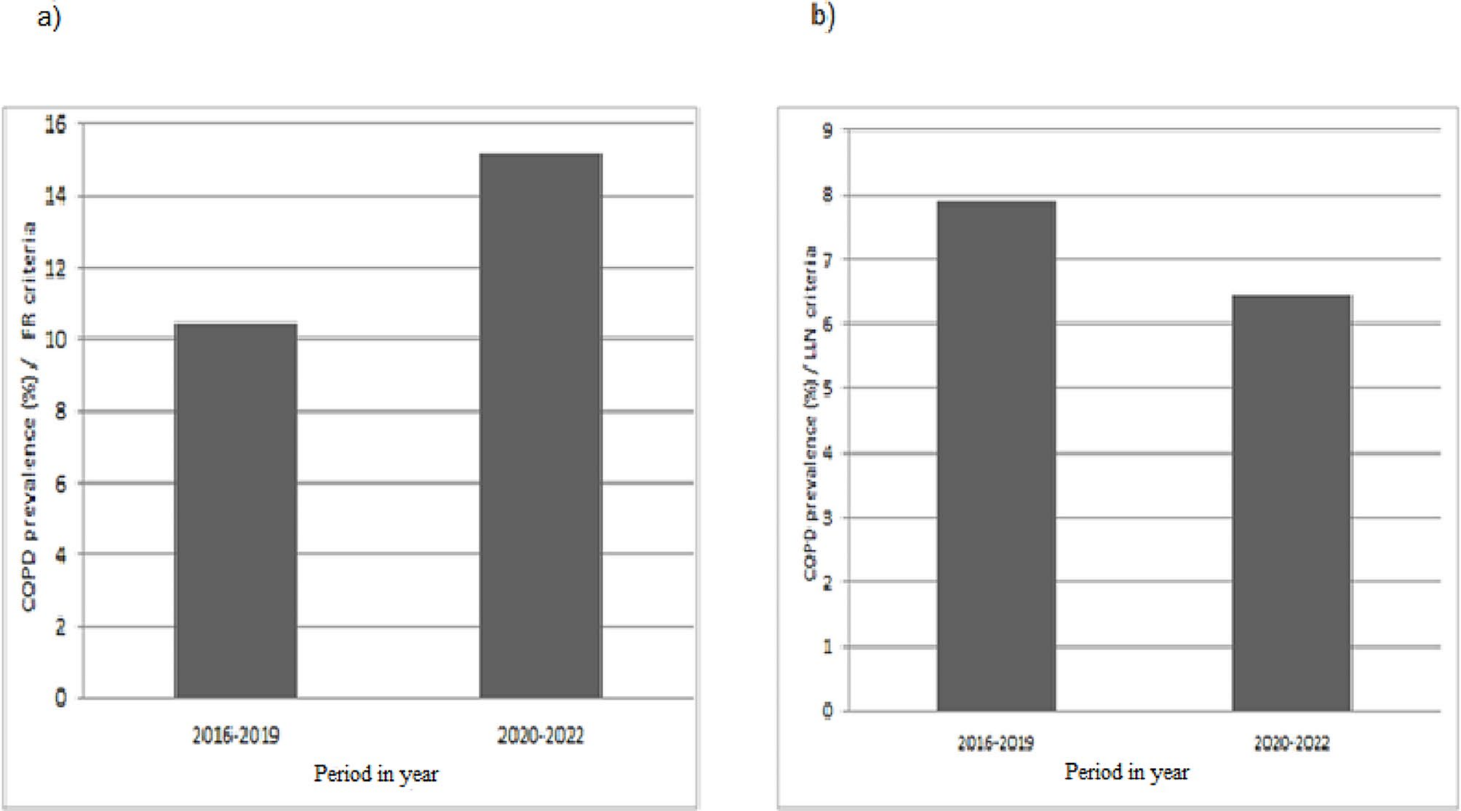
Estimated prevalence of COPD among individuals aged 40 years and older during 2016–2019 and 2020–2022. (**a**) Estimated prevalence of COPD among individuals aged 40 years and older during 2016–2019 and 2020–2022 by FR criteria. (**b**) Estimated prevalence of COPD between 2016–2019 and 2020–2022 by LLN criteria

The prevalence of COPD increased significantly between the 2016–2019 and 2020–2022 periods. According to the FR criteria, it was 10.43% (95% CI 8.11%-12.99%) in the 2016–2019 period and reached 15.17% (95% CI 11.67%-19.02%) in the 2020–2022 period (*P* < 0.001). Using the LLN as the diagnostic criteria, there was a significant but small decrease in the prevalence of COPD between the periods 2016–2019 and 2020–2022; it was 7.88% (95% CI 6.60% -9.27%) in the period 2016–2019 and 6.46% (95% CI 2.62%-11.84%) in the period 2020–2022 (*P* < 0.0001). (Figure 4, and Figure 1 of supplementary material)

### Bias of publication

**Fig. 5:**
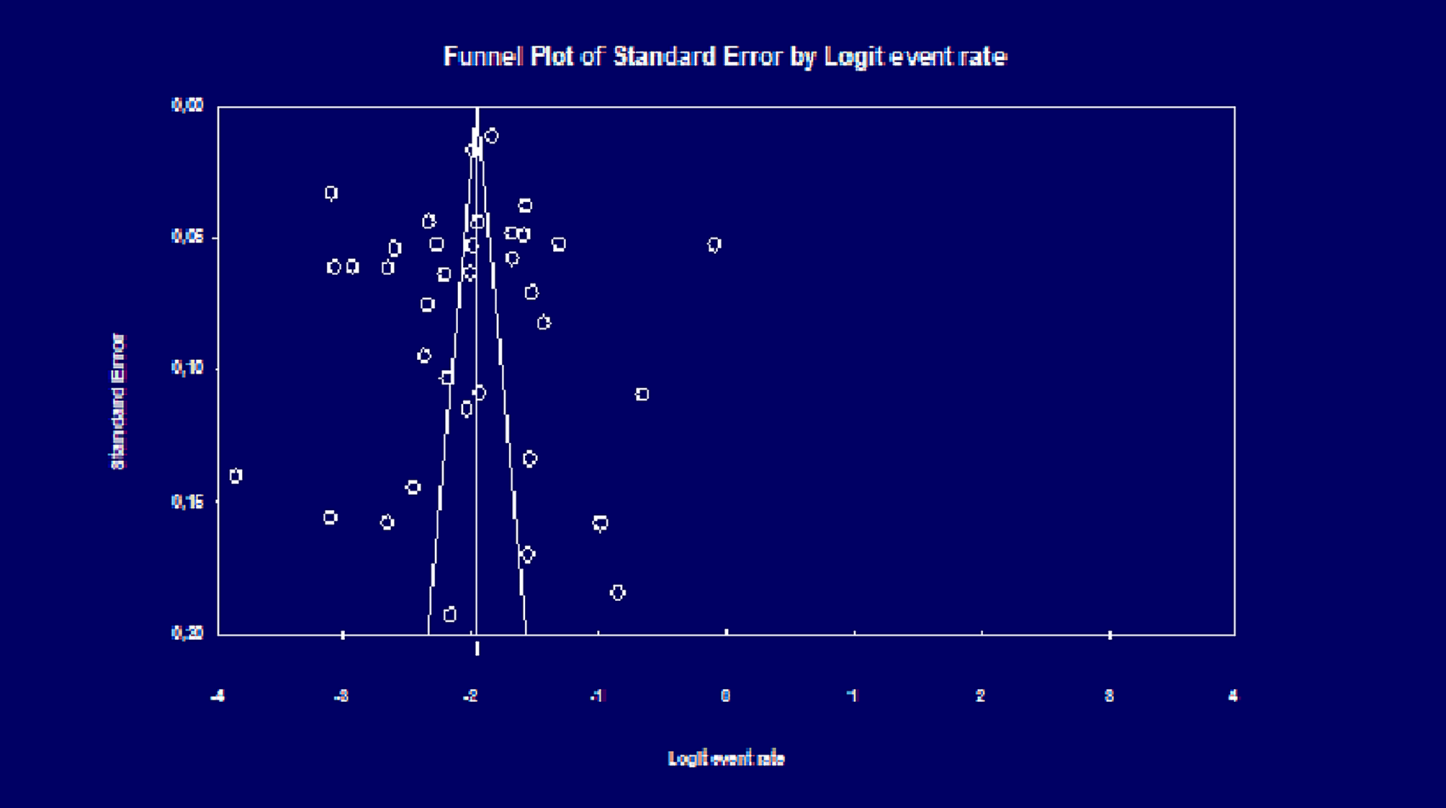
Funnel plot of a meta-analysis of studies on the global prevalence of COPD based on FR criteria

The limited number of studies did not allow to assess the bias of publication in some determinants. Whereas, these biases of publication were studies in some other determinants related to FR criteria (FR overall, age groups, residence, severity stage, sex, smoking status, 2016–2019 period, 2020–2022 period) and related to LIN criteria (LIN overall, sex, WHO region, 2016–2019 period). An asymmetric funnel plot was observed in all determinants, suggesting the existence of bias of publication between the included studies. The results of Egger’s test was confirmed the existence of these biases of publication (Fig. 5).

## Discussion

This review presents an estimate of the worldwide prevalence of COPD in people aged 40 years and older during the last 6 years, according to the most frequently used spirometric interpretation criteria, namely FR and LLN. Our study also aims at estimating the prevalence of COPD according to several parameters, such as age categories, smoking status, and severity stage, which will serve as a basis for understanding the burden of COPD and instituting effective prevention strategies.

The estimated overall prevalence of COPD in people aged 40 years and older was 12.64% (95% CI, 10.75-14.65%) and 7.38% (95% CI, 5.47-9.55%) according to the FR and LLN definitions, respectively. This estimate is similar to that reported by Vermaghani et al., who estimated an overall COPD prevalence of 12.16% (95% CI 10.91%-13.4%) according to the FR definition [19]. A previous meta-analysis that had as its main objective to estimate the overall prevalence of COPD in people aged 30 years and older during the period 1990–2010, according to the same diagnostic criteria, reported a prevalence of 11.7% [18], a lower estimate than that reported by our study. Furthermore, in a recently published meta-analysis, the worldwide prevalence of COPD according to the FR definition was 10.3%, which is lower than our results [20]. Even more, we found a prevalence of 15.17% (95% CI, 11.67-19.02%) during the period 2020–2022 according to the FR definition, which is much higher than that estimated by Adeloye et al. [20]. Consequently, the prevalence of COPD, according to FR criteria, is rising steadily, and targeted efforts to control this chronic respiratory condition are deemed necessary. The overall prevalence of COPD according to the LLN criteria is similar to that reported by Adeloye et al., who reported a total COPD prevalence of 7.6% [20]. Furthermore, comparison of COPD prevalence between the 2016–2019 period and the 2020–2022 period showed a slight decrease in COPD prevalence from 7.88 to 6.46% over the two periods, respectively. These data lead to the main conclusion that COPD prevalence estimates differ considerably depending on the diagnostic criteria used.

Estimates of the overall prevalence of COPD by sex indicate a high prevalence of irreversible airflow limitation in men compared to women according to the FR definition (prevalence of COPD was 15.47% in men versus a prevalence of 8.79% in women). This finding is supported by previous evidence showing a high prevalence of COPD among male participants, according to the same diagnostic criteria. For example, Vermaghani et al. estimated a prevalence of 15.70% and 9.93%, respectively, for men and women [19]. Furthermore, Adeloye et al. revealed that 14.3% of men aged 30 years and older suffer from COPD, compared to a prevalence of 7.6% in women [18]. A recent meta-analysis with the main objective of estimating the prevalence of COPD by sex revealed a synthetic prevalence of 8.16% in men and 6.16% in women [71]. The difference in prevalence between the two sexes according to the FR criteria could be explained by the fact that men consume more tobacco than women [72], and have a high risk of occupational exposure [73]. However, tobacco consumption by women in developed countries and the use of biofuels for cooking and heating by women in developing countries could decrease the gap observed in this study [74]. Using LLN as a diagnostic criteria, no difference in terms of COPD prevalence between the two sexes was observed (8.67% in men vs. 8.00% in women). A similar result was found by a previous study, which indicated that the prevalence of COPD according to LLN criteria did not differ between men and women [75].

Our meta-analysis found a high prevalence of COPD in the American region, with a prevalence of 22.93% according to the FR definition. This is similar to the results of many previous meta-analyses of regional COPD prevalence estimates. For example, Adeloye et al. and Vermaghani et al. reported that the American region had the highest prevalence compared to other regions, with prevalences of 15.2% and 14.53% respectively [18, 19]. Furthermore, comparison of regional estimates with those found in our study, indicates that the prevalence of COPD is increasing both globally and regionally. Our results also indicate that the lowest prevalence was recorded in the Eastern Mediterranean region (prevalence of 7.95% according to the FR definition). This differs from previous evidence, which found that the lowest prevalence was recorded in the South East Asian region [18, 19]. Using the LLN definition as the diagnostic criteria, the highest prevalence was recorded in the South East Asian region and the lowest in the Americas. This finding confirms that the spirometric definition used impacts the reported prevalence of COPD. Similarly, the heterogeneity of COPD prevalence between regions could be explained by the difference in associated risk factors, survey methodology, case definition used, as well as the characteristics of the included sample [76–78].

The contradictory results obtained in this systematic review and meta-analysis lead to a main conclusion, which stipulates that the spirometric criteria used to make the diagnosis affects the estimated prevalence of COPD and therefore makes comparisons difficult. Consulting the scientific literature, several studies have been carried out to propose which of the two definitions is the best for making an adequate diagnosis of COPD and have drawn sometimes a contradictory conclusions. Van Dijik and colleagues conducted a systematic review of the literature to compare the clinical relevance of the two diagnostic criteria and concluded that the severity criteria of airflow limitation can help in choosing which spirometric criteria to apply. The authors suggested using the FR criteria for the most severe cases and the LLN for the least severe [79]. In another meta-analysis, the authors compared the risk of comorbidities and mortality in patients with different diagnostic criteria and revealed a high risk of mortality in patients meeting both criteria and a risk of exacerbations in patients diagnosed by the FR [80]. In another study, Manino et al. showed that patients meeting LLN criteria were four times more likely to die [81]. Each criteria has its limitations. The FR overestimates COPD in the elderly and underestimates it in the young [76, 82, 83], leading to unnecessary treatment and healthcare expenditure [84]. On the other hand, the use of LLN as a diagnostic criteria can lead to different estimates of COPD depending on the LLN used [85]. Some researchers have also criticized the reference equations used, which do not incorporate all covariates [86]. In addition, Burney and colleagues revealed that defining disease according to reference values measured in a representative sample of the normal population biases the fraction attributable to the population [87]. Determining the best diagnostic criteria is therefore not possible, and the debate surrounding this topic is still open. An international consensus on the appropriate diagnostic criteria is needed to establish an accurate diagnosis and reduce the burden of this chronic disease worldwide.

We found that the most frequent COPD stage was the moderate COPD stage with a prevalence of 50.46%, followed by the mild COPD stage with a prevalence of 35.21%. The severe and very severe COPD stages were the least frequent, with a prevalence of 6.77% and 0.9%, respectively. This result is consistent with the findings of many previous studies. For example, Vermaghani and colleagues reported that the majority of COPD patients are in the moderate stage of the disease [19]. This highlights the need to promote early diagnosis and management of COPD patients in the less severe stages of the disease.

The existing literature indicates that COPD increases significantly with aging, this was confirmed by our meta-analysis. The prevalence of COPD according to the FR definition increased from 4.37 to 24.03% in people in the age group 40–49 years and those aged 70 years and over, respectively, and increased from 5.22 to 14.23% according to the LLN definition among the same age group, respectively. Indeed, age is an important risk factor that increases COPD morbidity and the risk of exacerbations in affected individuals [88, 89]. The model of Fletcher and Peto suggests that the rate of mean expiratory volume in one second (FEV1) decreases with age [90]. This model has subsequently been validated by other studies. For example, a prospective cohort study found that the annual rate of FEV1 decreased in people over 67 years of age than in people of younger age [91]. This could be explained by the fact that with aging, alveolar spaces widen and the lungs lose their elasticity, the risk of oxidative stress increases, and the number of anti-aging molecules decreases [92]. In light of these data, the government and public health policy makers should pay more attention to the elderly in order to detect COPD early and avoid any complications that may endanger the health and/or well-being of the elderly. We found that smoking was associated with a high prevalence of COPD. Indeed, smoking is a well-recognized risk factor for COPD [93, 94]. Tobacco smoke induces the proliferation of immune cells and the appearance of inflammatory mediators responsible for the lesions characteristic of COPD [95, 96]. Data have indicated that a reduction in tobacco consumption leads to a significant reduction in the number of COPD-related deaths. Therefore, tobacco control should be a global health priority for governments.

The present study has a number of limitations. Firstly, heterogeneity between studies was high, which could influence the interpretation of the results. In addition, we did not estimate the overall prevalence of pre- and post-bronchodilator COPD. A high number of the included studies estimated the pre-bronchodilator prevalence. Yet, guidelines recommend bronchodilator administration to establish the diagnosis of permanent flow obstruction and differentiate it from asthma. Therefore, the estimates revealed by the present study may not present the true prevalence of COPD. The distribution of studies across regions was disproportionate. Therefore, the regional prevalence reported in the present study may overestimate the burden of COPD in some regions and underestimate it in others. Furthermore, given the lack of published studies on COPD by FR criteria in Africa, we were unable to estimate the prevalence of COPD in this region. These data illustrate the enormous need for prevalence data in developing countries regions. We limited our search to articles published in English and French, which may miss the inclusion of other publications reporting prevalence data for this chronic respiratory condition in other languages. Similarly, the non-inclusion of COPD-related terms in the search strategy, such as chronic bronchitis or pulmonary emphysema, could miss capturing relevant studies. However, the estimation of the prevalence of COPD globally, regionally, and by several other parameters according to the two most widely used spirometry criteria was among the strengths of this meta-analysis.

## Conclusions

COPD is a significant public health problem. In this study, we found that the prevalence of COPD differs considerably depending on the diagnostic criteria used. Alarming data on the prevalence of COPD by several parameters were identified and were consistent with existing evidence. Therefore, the control of COPD must be a major health concern of public authorities in order to reduce the global burden of this chronic respiratory condition. This cannot be achieved in the absence of effective management and prevention strategies targeting the risk factors involved in the development of permanent airway obstruction.

### Electronic supplementary material

Below is the link to the electronic supplementary material.

Supplementary Material 1

## Data Availability

All data generated or analysed during this review are included in this published article and its supplementary information files.

## Abbreviations

COPD: Chronic obstructive pulmonary disease
FR: Fixed ratio
LLN: Lower limit of normal
FVC: Forced vital capacity
FEV 1: Forced expiratory volume in one second
WHO: World health organazation
GBD: Global burden of disease
PRISMA: Preferred reporting items for systematic reviews and meta-Analyses
STROBE: Strengthening the reporting of observational studies in epidemiology
EMR: Eastern Mediterranean Region
EUR: European Region
AMR: Americas Region
SEAR: South East Asia Region
WPR: Western Pacific Region
AFR: African Region
